# Feasibility and safety of electrochemotherapy (ECT) in the pancreas: a pre-clinical investigation

**DOI:** 10.1515/raon-2015-0013

**Published:** 2015-03-25

**Authors:** Roberto Girelli, Simona Prejanò, Ivana Cataldo, Vincenzo Corbo, Lucia Martini, Aldo Scarpa, Bassi Claudio

**Affiliations:** 1 Pancreatic Unit – Casa di Cura Pederzoli, Peschiera del Garda (VR), Italy; 2 ARC-NET Research Centre and Department of Pathology and Diagnostics, University and Hospital Trust of Verona, Verona, Italy; 3 Laboratory of Preclinical and Surgical Studies and Laboratory of Biocompatibility, Innovative Technologies and Advanced Therapies, Rizzoli Orthopedic Institute Bologna, Italy; 4 Department of Surgery and Oncology, Pancreas Institute, University and Hospital Trust of Verona, Verona, Italy

**Keywords:** electroporation, bleomycin, cisplatin, electrochemotherapy, preclinical study, safety, pancreatic adenocarcinoma

## Abstract

**Background.:**

Pancreatic ductal adenocarcinoma (PDAC) is a lethal disease generally refractory to standard chemotherapeutic agents; therefore improvements in anticancer therapies are mandatory. A major determinant of therapeutic resistance in PDAC is the poor drug delivery to neoplastic cells, mainly due to an extensive fibrotic reaction. Electroporation can be used *in vivo* to increase cancer cells’ local uptake of chemotherapeutics (electrochemotherapy, ECT), thus leading to an enhanced tumour response rate. In the present study, we evaluated the *in vivo* effects of reversible electroporation in normal pancreas in a rabbit experimental model. We also tested the effect of electroporation on pancreatic cancer cell lines in order to evaluate their increased sensitivity to chemotherapeutic agents.

**Materials and methods.:**

The application *in vivo* of the European Standard Operating Procedure of Electrochemotherapy (ESOPE) pulse protocol (1000 V/cm, 8 pulses, 100 μs, 5 KHz) was tested on the pancreas of normal New Zealand White Rabbits and short and long-term toxicity were assessed. PANC1 and MiaPaCa2 cell lines were tested for *in vitro* electrochemotherapy experiments with and without electroporation. Levels of cell permeabilization were determined by flow cytometry, whereas cell viability and drug (cisplatin and bleomycin) sensitivity of pulsed cells were measured by 3-(4,5-dimethylthiazol-2-yl)-5-(3-carboxymethoxyphenyl)-2-(4-sulfophenyl)-2H-tetrazolium (MTS) assay.

**Results.:**

In healthy rabbits, neither systemic nor local toxic effects due to the electroporation procedure were observed, demonstrating the safety of the optimized electric parameters in the treatment of the pancreas *in vivo*. In parallel, we established an optimized protocol for ECT *in vitro* that determined an enhanced anti-cancer effect of bleomycin and cisplatin with respect to treatment without electroporation.

**Conclusions.:**

Our data suggest that electroporation is a safe procedure in the treatment of PDAC because it does not affect normal pancreatic parenchyma, but has a potentiating effect on cytotoxicity of bleomycin in pancreatic tumour cell lines. Therefore, ECT could be considered as a valid alternative for the local control of non-resectable pancreatic cancer.

## Introduction

Pancreatic ductal adenocarcinoma (PDAC) is a highly aggressive disease with a poor 5-year survival, resulting in the fifth leading cause of cancer-related death in Europe.^1^–^3^ PDAC is usually diagnosed in advanced stage, with loco-regional invasion and distant metastasis, and usually results largely drug-resistant. Surgical resection, although is currently the only “curative” chance to prolong survival, is suitable only for a minority of patients (< 20%). Standardized protocols of treatment in resectable patients provide a 6-month-course adjuvant chemotherapy following resection^4^, although the survival is still poor.

The optimal treatment for patients with locally advanced pancreatic cancer (LAPC) with no evidence of metastases remains to be defined. The standard treatment for LAPC is based on Chemotherapy (CT)^5^–^9^ and combination of CT and Radiotherapy (RT) with a median overall survival of 10–15 months.^10^–^12^

Because of the poor results achieved, some new ablative techniques have been considered for local treatment: radiofrequency ablation (RFA), laser ablation, microwave, ethanol injection and irreversible electroporation (IRE).^13^ Particularly, RFA has been used in the clinical setting with promising preliminary results.^14^–^17^

Patients with metastatic disease undergo systemic palliative chemotherapy. Treatment outcomes are typically poor and resistance to standard chemotherapeutic agents remains the major problem.^18^–^24^

Based on recent preclinical data in mouse models, it has been hypothesised that currently available anticancer drugs cannot access tumour cells at an effective concentration due to the presence of an extensive hypo-vascular fibrotic stroma that acts as a barrier shielding tumour cells from the action of systemic therapeutic agents.^25^ Therefore, the improvement of anticancer drug delivery enhances tumour cells sensitivity and hence would provide a better response and a potential prolongation of survival.

Electroporation is a physical method that employs microsecond length electric pulses to alter temporarily the permeability of cell membranes in order to facilitate chemicals and large molecules delivery to the cells.^26^–^28^ Electroporation can be used in all types of isolated cells as well as in tissues. Target cells have to be exposed to an electric field of sufficient strength and for a sufficient time. The magnitude of the electric field depends on cell type, size, orientation and density, pulse duration and number of pulses.^29^

Electrochemotherapy (ECT) combines the administration of non-permeant or low-permeant cytotoxic drugs (bleomycin or cisplatin) with the application to the tumour of permeabilizing electric pulses, to increase local drug uptake and hence its effectiveness.^30^, ^31^

This combination treatment has proven to be very effective in local control of skin metastatic tumour nodules independently of the histotype^32^–^39^ and it is now routinely employed for this purpose in a number of European countries, in about 140 cancer treatment centres; recently this technique has been developed for the treatment of deep-seated tumours.^40^,^41^

Electrochemotherapy has been used in previous preliminary studies for the treatment of rats and mice bearing subcutaneous implants of melanoma^30^,^41^–^44^, sarcoma^30^,^43^–^45^, and pancreatic tumours^46^ with encouraging results. Furthermore, liver tumours of rats and rabbits resulted highly responsive to ECT without impairment of the surrounding normal tissue functionality.^47^–^48^ The effectiveness of intraoperative ECT was demonstrated in hamster pancreatic adenocarcinoma cell line (PC-1) model.^49^

We hypothesize that ECT could be used for the treatment of primary non-resectable pancreatic cancer. The aim of the present study was to investigate the feasibility and safety of electroporation in an experimental rabbit model. *In vivo* local and systemic effects of electric pulses applied to the pancreas were evaluated. Furthermore, we tested ECT *in vitro* on highly drug resistant pancreatic cancer cell lines in order to prove its efficacy in this subset as well.

## Materials and methods

### Electroporation apparatus

Electroporation was performed using the Cliniporator^™^ (IGEA S.p.A., Carpi, Modena, Italy). Different protocols of 100 μs square-wave electric pulses were tested *in vitro*; one sequences of 8 electrical pulses, 100 μs of duration, at 1000 V/cm were used for *in vivo* experiments.

### Animals and anaesthesia

The experiments were conducted according to the EU Directive 2010/63/EU and to the Guidelines for the welfare and use of animals in cancer research (Br J Cancer 2010; 102:1555–77). The protocol was approved by the Ethical Committee of the Rizzoli Orthopaedic Institute (ELETTRO-RAB 03/25/2009 prot.10499), and submitted to the Italian Ministry of Health (28/04/2009 prot.10771). We used 9 male Hybrid New Zealand rabbits weighing 2.1 ± 0.5 kg, housed under controlled conditions. General anaesthesia was induced with an intramuscular injection of 44 mg/kg ketamine (Imalgene 1000, Merial Italia S.p.a., Milano), 3 mg/kg xylazine (Rompun: Bayer S.p.a. Milano) and maintained by means of facial mask in spontaneous ventilation (O_2_: 1 L/min, N_2_O: 0.4 L/min, isoflurane: 2.5% to 3%). Postoperatively, antibiotic and analgesic therapy was administered (Flumequine - Flumexil Fatro, Italy and metamizole Farmolisina Vetem, Italy).

General physiological status of rabbits, including potential onset of anorexia and intestinal blocking, was monitored daily by technician and in charge veterinarian during all the period of the study.

### Animal electroporation and sample collection

Animals underwent pancreas and duodenum (gut) electroporation in open surgery according to the standard operating procedure: 8 pulses at 1000 V/cm of amplitude over distance ratio, 100 μs of duration were delivered at 5 KHz using linear N-20-4B electrodes. The electroporated area ranged between 12–15 mm in length. The animals were euthanized 24 hours (n = 2), 72 hours (n = 1), 15 days (n = 3), 30 days (n = 3) after surgery with *i.v.* injection of 2 ml Tanax (Tanax, Intervet International AN Baxmeer NL) under deep general anaesthesia. The pancreas was removed and treated for the morphological and histological analysis. All the specimens were formalin fixed and paraffin embedded. Four mm tissue sections were stained with Haematoxylin and Eosin (H&E) for histopathological evaluation. Post mortem bowel was visually explored for integrity. No histological evaluation was performed on the bowel.

### Biochemical analysis

Peripheral blood was collected before surgery (T0) and at each experimental time: 7 days, 15 days and 30 days following electroporation. After blood collection, serum was separated by centrifugation and parameters of hepatic function such as alanine transaminase (ALT), aspartate transaminase (AST), and pancreatic enzymes, such as amylase, were analysed. The animals did not receive any pharmacological prophylaxis for pancreatitis such as octreotide and Gabesato mesylate. Since the *in vivo* study is limited to tissue electroporation and no drug was administered to the animals, for ethical reasons, we did not include control groups.

### Cell line and drugs

PANC1 and MiaPaCa2 human pancreatic cancer cell lines were obtained from the American Type Culture Collection (ATCC). Various molecular mechanisms have been taken in account to play a role in drug-resistance development in these specific cell lines. One of these mechanisms is mediated by the overexpression of BRG1, a chromatin modulator responsible for gemcitabine resistance also in locally advanced and metastatic pancreatic cancer.^50^

The two cell lines were cultured in RPMI 1640 medium supplemented with 10% of heat-inactivated foetal calf serum (Gibson, Milan, Italy). Cells were maintained as monolayer in 75 cm^2^ tissue culture flasks at 37°C in a humidified atmosphere of 5% CO_2_. The cell lines were Mycoplasma free as assessed by MycoAlert assay (Lonza, Milan, Italy). Cells used for experimental purposes were detached using trypsin-EDTA (0.05% trypsin, Lonza) and collected by centrifugation at 300 rpm. Cell diameter for PANC1 and MiaPaCa2 was 16.8 μm and 12.2 μm respectively, as measured by FACS analysis. The following anticancer drugs were used: bleomycin and cisplatin (Sigma, Milan, Italy). Drugs were dissolved in 0.9% bacteriostatic saline and used at indicated concentrations.

### Cell viability and permeabilization

Three aliquots of 1×10^5^ cells (in 100μl of RPMI medium) were placed in separate 2 mm gap electroporation cuvettes (Bio-Rad laboratories). Each cuvette was exposed to electric field strength of 0.5 or 1 kV/cm (8 pulses, 100 μs of duration, 5 KHz). After 30 minutes of incubation, pulsed cells were diluted by a factor 100 in RPMI medium and seeded into 96-well tissue culture plates. Unpulsed cells were subjected to same procedure. The acute effect of electroporation on cell viability was determined using MTS assay (Promega) and the Countess Cell Counter (Invitrogen, Milan, Italy). Cell permeabilization was determined at indicated field strength by uptake of fluorescent dye Lucifer yellow (Sigma, Milan, Italy). Briefly, cells incubated in the presence of Lucifer yellow at a final concentration of 1 mM were exposed to electric pulses and then incubated at 37°C for 5 min. Cells were then chilled on ice, washed in phosphate-buffered saline (PBS) buffer and examined for fluorescence emission in a FACScalibur flow cytometer (Beckton Dickinson). Median fluorescence emission was determined and percent of permeabilized cells was calculated.

### *In vitro* electrochemotherapy (ECT)

Cell sensitivity of PANC1 and MiaPaCa2 to cisplatin and bleomycin was preliminary tested in order to define an appropriate range of drug concentrations to be used for electrochemotherapy experiments (data not shown). For ECT, cells (1×10^5^ suspended in 100μl of RPMI medium) were placed between 2 plate electrodes in 2 mm gap cuvette (Bio-Rad laboratories) in the presence of a range of concentration of drugs or isotonic saline and exposed to a defined electric field strength of 1 kV/cm (8 pulses, 100 μs of duration). Aliquots of cells were left unpulsed in presence of drugs or vehicle. Seven concentrations of cisplatin ranging from 1.6 to 26 μM were used for PANC1, whereas 7 concentrations ranging from 1.6 to 40 μM were tested for MiaPaCa2. For bleomycin, 5 concentrations ranging from 0.1 to 6.6 μM were used for MiaPaCa2, whereas 7 concentrations ranging from 0.3 to 130 μM were tested for PANC1. Pulsed and unpulsed cuvettes were incubated in humidified atmosphere at 37°C for 30 min after electric exposure. Then cells were diluted by a factor of 100 in RPMI medium and three aliquots of 100 μl (1×10^4^ cells) from each cuvette were seeded in 96-well tissue culture plates. Plates were incubated for 72 h and then survival was evaluated by MTS assay (Promega) and the Countess Cell Counter (Invitrogen, Milan, Italy).

Data from the plate reader were analysed using GraphPad Prism version 6. The data were first normalized to the vehicle control and then analysed using the nonlinear regression feature. The curves shown in the figures and the calculated IC50 values were the result of three technical replicates for each cell lines.

### Statistical analysis

Comparisons between groups were performed using Student’s T test and p < 0.05 level was considered significant.

## Results

### *In vivo* electroporation of non-pathological rabbit pancreata; general toxicity and histological evaluation

**FIGURE 1.**
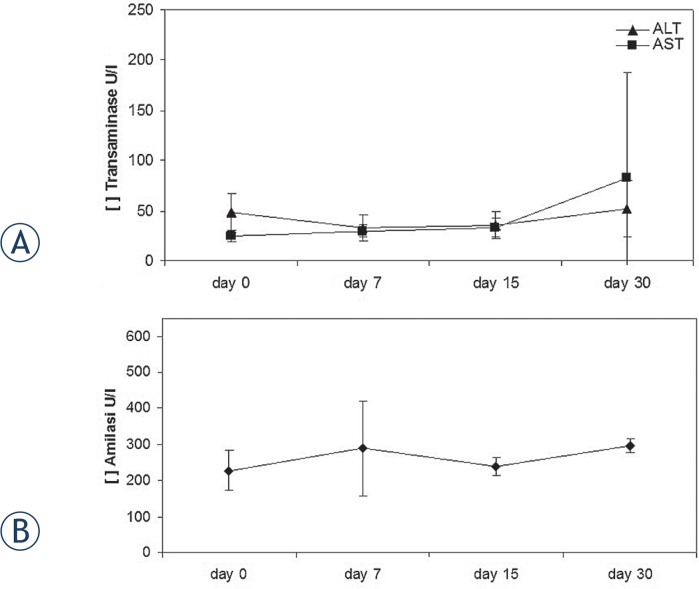
Serum levels of the liver enzymes, AST, ALT **(A)** and of the pancreatic amylase **(B)**. Data points represent mean ± (standard error) SE, n = 3–6.

General physiological status of the rabbits was checked daily and no functional deficits as anorexia, intestinal blockage or other clinical status alterations were detected; all the rabbits recovered from general anaesthesia after 1 hour. Transaminase and amylase levels were not statistically modified (p > 0.05) over 30 days of the electroporation procedure (day 0, day 7, day 15, day 30) (Figure 1).

### Histological evaluation

**FIGURE 2.**
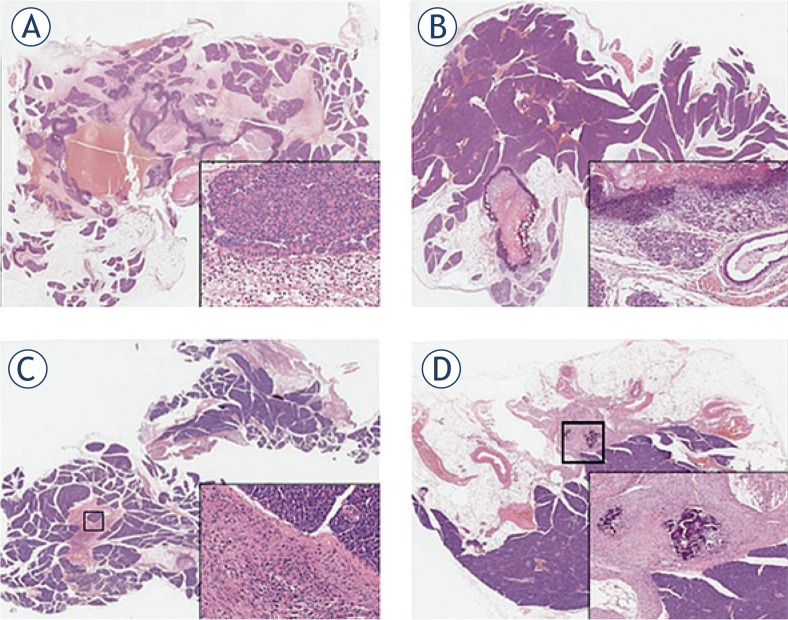
Photomicrographs of rabbit pancreata at different times after electroporation. **(A)** 24 hours: necrosis in the treated areas surrounded by significant hyperaemia and oedema. Acinar degranulation and vacuolation with granulocytes and lymphocytes infiltration (inset). **(B)** 72 hours: necrosis is still evident while acinar to ductal metaplasia appears along with intraductal protein plugs (inset). **(C)** 15 days: fibrotic areas are evident; chronic inflammatory cells are present while pancreatic acini are normal (inset). **(D)** 30 days: calcium deposition is detectable in fibrotic areas; pancreatic parenchyma is normal (inset).

The results of hystopathological analysis are shown in Figure 2A–D. At short term, 24 h post-electroporation, non-pathological rabbit pancreas showed necrosis in the treated areas surrounded by important hyperaemia and oedema in addition to aspects of acute distress of acinar cells (degranulation and vacuolation). An acute inflammatory reaction was also present (Figure 2A). Acinar to ductal metaplasia appeared after 72 h. Elevation of serum amylase is often, but not always, associated with acute pancreatitis. In our study, we found that the ductal metaplasia is not associated with an increase in the serum level of amylase. It is possible that the ductal metaplasia is the consequence of the transient inflammatory response to the insertion of the needle rather than the inflammatory response of the entire organ.

Moreover, the evidence of intraductal protein plugs, containing degenerating cells, was due to proteins hypersecretion by acinar cells (Figure 2B). After 15 days, fibrotic areas surrounded by normal pancreatic acinar cells were detected. Fat substitution of about 20% of pancreatic parenchyma was also present (Figure 2C). After 30 days, calcium deposition was evident in fibrotic areas (Figure 2D) as a result of mild and transient modification.

When electrical pulses were directly applied to the gut, its integrity was maintained without infection on further clinical complications (data not shown).

### Cells survival and permeabilization

**FIGURE 3.**
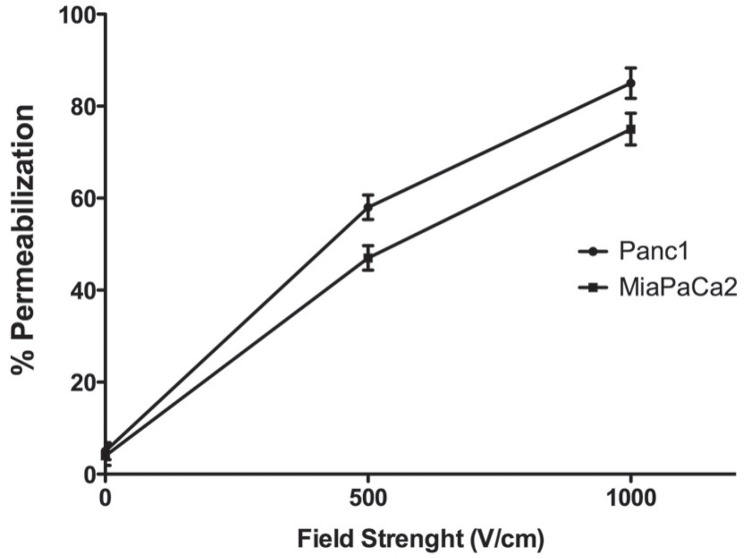
Cell lines permeabilization as determined by Lucifer yellow uptake and flow cytometry. Mean ± standard deviation (S.D.) of n = 3 independent experiments; circle symbol, Panc1; square symbol, MiaPaCa2

Before testing the toxicity of anticancer drugs on electropermeabilized cells, we defined the optimal parameters for electrical treatment in order to obtain high cell survival and effective permeabilization. Therefore, both PANC1 and MiaPaCa2 cells were subjected to 8 pulses of 100 μs at two different electric field strength of 0.5 or 1 kV/cm. Percent of viable cells was calculated with respect to untreated cells. Field strength of 0.5 kV/cm did not affect cell viability, whereas a slight reduction of about 4% was observed when both cell lines were exposed to field strength of 1 kV/cm (data not shown). Cell permeabilization was assessed by flow cytometry at 1 kV/cm showing that about 90% and about 75% of cells were permeabilized, for PANC1 and MiaPaCa2 respectively (Figure 3). The optimal electric condition was defined as 1 kV/cm, 8 pulses of 100μs duration. In these conditions, the mean value of viability of the pulsed controls was 95% (range 93–97%) of that of the unpulsed controls, with a permeabilization level of at least 75% of pulsed cells.

### Cell viability following electrochemotherapy

**FIGURE 4.**
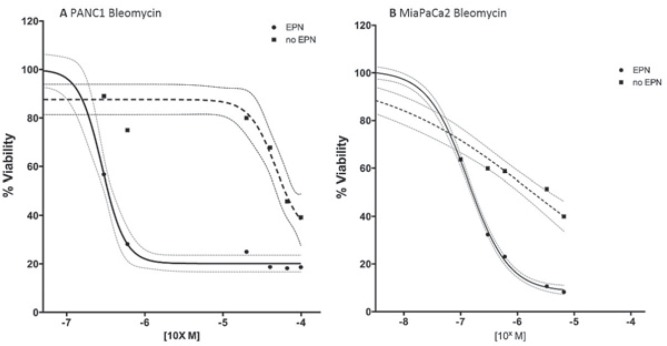
Dose-response curve of bleomycin treatment. Cell viability was assessed at 72 hours of drug exposure using 3-(4,5-dimethylthiazol-2-yl)-5-(3-carboxymethoxyphenyl)-2-(4-sulfophenyl)-2H-tetrazolium (MTS) assay (Promega) and the Countess Cell Counter (Invitrogen, Milan, Italy). Dashed line = no electroporation; solid line = electroporation; grey lines indicate 95 % confident interval [CI]

**FIGURE 5.**
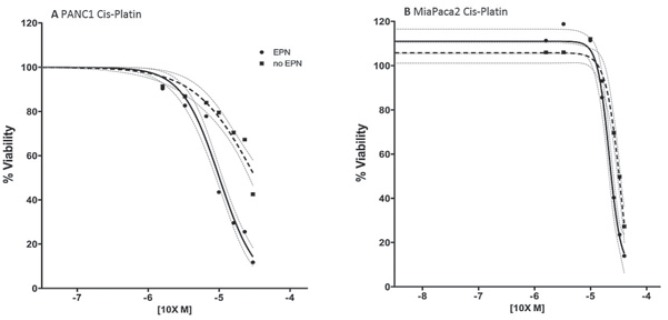
Dose-response curve of cisplatin treatment. Cell viability was assessed at 72 hours of drug exposure using 3-(4,5-dimethylthiazol-2-yl)-5-(3-carboxymethoxyphenyl)-2-(4-sulfophenyl)-2H-tetrazolium (MTS) assay (Promega) and the Countess Cell Counter (Invitrogen, Milan, Italy). Dashed line = no electroporation; solid line = electroporation; grey lines = indicate 95% confident interval [CI]

After establishing the optimized parameters for electroporation, 8 pulses of 100μs duration at 1 kV/cm, we investigated the cytotoxicity of two chemotherapeutic agents, bleomycin and cisplatin, against PDAC cell lines. Pulsed PANC1 and MiaPaCa2 cell lines showed enhanced sensitivity to both bleomycin and cisplatin compared to unpulsed cells. Specifically, the IC50 of bleomycin for PANC1 and MiaPaCa2 was reduced by a factor 166 and 18 respectively, after electroporation. Whereas the IC50 of cisplatin was reduced by a factor 2.8 for PANC1 and 1.3 for MiaPaCa2 (Table 1). The cells viability after bleomycin and cisplatin treatment pulsed with electroporation and unpulsed (Figure 4,5).

## Discussion

In this study, we demonstrated that a well-defined electroporation protocol does not induce evident signs of local and systemic toxicity when applied to normal pancreas in a pre-clinical model. In addition, the effect of ECT with cisplatin and bleomycin was evaluated in human pancreatic cancer cells lines after. ECT refers to the combined administration of chemotherapy with the local application of electric pulses to the tumour cells in order to increase drug delivery and local cytotoxicity. ECT has been proven effective in the treatment of skin or subcutaneous metastases from solid tumours of different origin. Subsequently to several reports of clinical studies^35^–^38^ assessing the use and the efficacy of ECT in the treatment of various primary skin cancers, head and neck cancer, and skin metastasis of different primary tumours, clinicians and researchers are trying to develop novel approaches to extend ECT to the treatment of deep-seated and visceral tumours.^39^–^41^ Edhemovic *et al*., have recently reported for the first time, the feasibility and safety of the procedure highlighting the effectiveness of ECT in the treatment of patients with liver metastases from primary colorectal cancer. The lack of side effect during and after the procedure, demonstrates the safety of the treatment.^41^ Furthermore, Granata *et al.* reported a preliminary experience of feasibility and safety of intraoperative electrochemotherapy in locally advanced pancreatic tumour. Twelve patients with tumours of the head or the body of the pancreas underwent ECT. No side effects or major complications have been recorded. No acute intraoperative or postoperative serious adverse effects were related to ECT, showing that electrochemotherapy is a feasible and safe treatment for patients with locally advanced pancreatic adenocarcinoma.^51^

According to the results reported in further studies, the viability of human tumoural pancreatic cell lines was not modified by electrical pulse alone, while it decreased after exposure to bleomycin and cisplatin. Bleomycin and cisplatin result cytotoxic only at high concentrations. Nevertheless, when the cells were exposed to both the chemotherapeutic agent and the electric pulses, the cytotoxic effect was achieved at lower drug concentration. Specifically, the potentiating effect of electric pulse was more pronounced for bleomycin than cisplatin, confirming previous observations.^35^,^41^,^49^,^52^–^54^ Todorovic *et al.*^55^ observed a similar sensitization effect in murine colon carcinoma cell-line CMT3 and reported the referral range of IC50 value of different murine and human cell lines treated with bleomycin and cisplatin, with and without electroporation. The pancreatic cell lines PANC1 and MiaPaCa2 tested in the present study, demonstrate IC50 values within the range indicated by Todorovic *et al.*

The delivery of electric pulses through needles inserted into the pancreas of rabbits elicits a local inflammatory response in the early days after ECT and completely resolves in 30-days. The blood values of pancreatic amylase and transaminases (ALT and AST) were not significantly increased over the whole observational period. Our data agree with previous results reported by Ramirez LH *et al*., which demonstrate that tissues submitted to electroporation alone, present an immediate inflammatory reaction restricted to electropulsed areas. No diffuse damage of adjacent organs was observed.^48^ Similarly, Cemazar M *et al*. analysed *in vivo* the ECT antitumour efficacy in a number of animal models.^56^ Our data confirmed that electroporation is a safe procedure in the treatment of pancreatic tumours and ECT could be effective for local control of non-resectable pancreatic cancer. The development of new electrodes and specific software for the assessment of proper preoperative strategies could increase both the safety of ECT and extend its application field.

## Figures and Tables

**TABLE 1.** IC50 of bleomycin and cisplatin for PANC1 and MiaPaCa-2 unpulsed (−) or pulsed (+) with electroporation (EP)

	**IC 50 of Bleomycin**			**IC 50 of Cisplatin**		

Cell	− EP	+ EP	P value	− EP	+ EP	P value
PANC1	100 μM	0.59 μM	<0.0001	23 μM	8 μM	<0.0001
MiaPaCa2	3.5 μM	0.2 μM	<0.0001	30 μM	23 μM	0.001
